# Wedelolactone Attenuates Pulmonary Fibrosis Partly Through Activating AMPK and Regulating Raf-MAPKs Signaling Pathway

**DOI:** 10.3389/fphar.2019.00151

**Published:** 2019-03-05

**Authors:** Jin-yu Yang, Li-jun Tao, Bei Liu, Xin-yi You, Chao-feng Zhang, Hai-feng Xie, Ren-shi Li

**Affiliations:** ^1^State Key Laboratory of Natural Medicines, School of Traditional Chinese Medicine, China Pharmaceutical University, Nanjing, China; ^2^Chengdu Biopurify Phytochemicals Ltd., Chengdu, China

**Keywords:** *Eclipta prostrata*, wedelolactone, pulmonary fibrosis, AMPK, bleomycin

## Abstract

Pulmonary fibrosis is common in a variety of inflammatory lung diseases, there is currently no effective clinical drug treatment. It has been reported that the ethanol extract of *Eclipta prostrata L*. can improve the lung collagen deposition and fibrosis pathology induced by bleomycin (BLM) in mice. In the present study, we studied whether wedelolactone (WEL), a major coumarin ingredient of *E. prostrata*, provided protection against BLM-induced pulmonary fibrosis. ICR or C57/BL6 strain mice were treated with BLM to establish lung fibrosis model. WEL (2 or 10 mg/kg) was given daily via intragastric administration for 2 weeks starting at 7-day after intratracheal instillation. WEL at 10 mg/kg significantly reduced BLM-induced inflammatory cells infiltration, pro-inflammatory factors expression, and collagen deposition in lung tissues. Additionally, treatment with WEL also impaired BLM-induced increases in fibrotic marker expression (collagen I and α-SMA) and decrease in an anti-fibrotic marker (E-cadherin). Treatment with WEL significantly prevented BLM-induced increase in TGF-β1 and Smad2/3 phosphorylation in the lungs. WEL administration (10 mg/kg) also significantly promoted AMPK activation compared to model group in BLM-treated mice. Further investigation indicated that activation of AMPK by WEL can suppressed the transdifferentiation of primary lung fibroblasts and the epithelial mesenchymal transition (EMT) of alveolar epithelial cells, the inhibitive effects of WEL was significantly blocked by an AMPK inhibitor (compound C) *in vitro*. Together, these results suggest that activation of AMPK by WEL followed by reduction in TGFβ1/Raf-MAPK signaling pathways may have a therapeutic potential in pulmonary fibrosis.

## Introduction

*Eclipta prostrata* L. is widely used to treat respiratory diseases such as diphtheria, pertussis, tuberculosis in the traditional medicine of China (Roy et al., 2008; Deng and Fang, 2012), which exhibits hepatoprotective (Tabassum and Agrawal, 2004; Manvar et al., 2012), anti-tumor (Liu et al., 2012) and other biological activities (Tewtrakul et al., 2011; Jaiswal et al., 2012). In Brazil, extracts of *E. prostrata* are also used to treat asthma (Chichioco-Hernandez and Paguigan, 2010; Sharma et al., 2012; Jahan et al., 2014). It has been reported that the methanol extract of this plant significantly attenuated experimental pulmonary fibrosis in mice (You et al., 2015). Although it has been found that WEL, a main component of *E. prostrata*, can improve bronchial epithelial cell injury (Ding et al., 2015) and fibrosis process of activated hepatic stellate cells (Xia et al., 2013), its effects on pulmonary function, collagen deposition and epithelia-mesenchymal transition remain to be researched.

Pulmonary fibrosis is a chronic inflammatory interstitial lung disease. Recently, several tyrosine kinase receptors, such as nintedanib (BIBF 1120), has been approved for treatment of PF (Myllärniemi and Kaarteenaho, 2015), but its potential side effects are still unknown. Recently, researchers have identified the close relationship between AMPK activation and lung fibrogenesis (Sato et al., 2016; Rangarajan et al., 2018). The administration of WEL can attenuate hepatic steatosis in mice by activating AMPK (Zhao Y. et al., 2015), but the therapeutic effect of WEL on pulmonary fibrosis is not sure. The processes of normal lung repair after injury include epithelial cell migration, proliferation and differentiation, lung fibroblast migration, and transformation of lung fibroblast into myofibroblasts (Selman and Pardo, 2001). The fibrotic response is driven by abnormally activated alveolar epithelial cells resulting in epithelial to mesenchyme transition (EMT) and formation of myofibroblast foci secreting amounts of ECM (Ley et al., 2011; Wynn, 2011).

Suppressing the activation of fibroblasts can ameliorate pulmonary fibrogenesis (Postlethwaite et al., 2004). TGF-β1 is the main cytokine in pulmonary fibrosis pathogenesis, which regulates fibroblasts proliferation and differentiation leading to ECM over-production (Sime et al., 1997; Khalil et al., 2001). BLM (an anti-neoplastic agent) causes alveolar cell damage, inflammatory response, EMT and subsequent ECM deposition to induce lung injury and pulmonary fibrosis *in vivo* (Gong et al., 2005). In the present study, the administration of WEL effectively attenuated BLM-induced pulmonary fibrosis process in mice by activating AMPK to negatively regulate collagen production and transformation of lung fibroblast into myofibroblasts.

## Materials and Methods

### Chemicals and Reagents

Wedelolactone (Pubchem CID: 5281813, purity above 99%) was prepared by Mr. Haifeng Xie in Chengdu Biopurify Phytochemical Ltd. (Chengdu, China). Prednisone acetate (PNS, Pubchem CID: 91438) was purchased from Zhejiang Xianju Pharmaceutical Co., Ltd. (Xianju, China). Bleomycin hydrochloride (BLM) was purchased from Nippon Kayaku (Tokyo, Japan). Compound C (Pubchem CID: 11524144), an AMPK inhibitor, was purchased from Shanghai Chembest Research Laboratories Limited (Shanghai, China). Recombinant TGF-β1 was purchased from PeproTech (Rocky Hill, NJ, United States). 3-(4,5-dimethylthiazol-2-yl)-2,5-diphenyl tetrazolium bromide (MTT) was purchased from Biosharp (Anhui, China).

Hydroxyproline assay kit was purchased from Beyotime Biotechnology (Jiangsu, China). Antibodies against ERK (^#^4695), phospho-ERK (^#^4370), JNK (^#^9258), phospho-JNK (^#^9255), p38 (^#^8690), phospho-p38 (^#^4511), AMPK (^#^2531), phospho-AMPK (^#^2532) and TGF-β (^#^3711) were all purchased from Cell Signal Technology Inc. (Danvers, MA, United States). Antibodies against COLI (WL0088), Raf1 (WL00553), and Vimentin (WL01960) were all obtained from Wanleibio (Shenyang, China). Antibodies against α-SMA (ab32575) was obtained from Abcam (Cambridge, United Kingdom). Antibodies against E-cadherin (BS72286) was obtained from Bioworld Technology Inc. (Dublin, OH, United States). HRP-conjugated secondary antibody was purchased from Bioworld Technology Inc. (Dublin, OH, United States).

### Cell Culture

Primary lung fibroblasts (PLFs) were derived from 6 to 8 weeks old male C57/BL6 mice. The lungs were cleaned in phosphate-buffered saline (PBS), minced into 1–2 mm^3^ sections and digested with trypsin for 30 min at 37°C. The cell suspensions obtained after digestion were plated into sterile cell culture bottle containing 5–6 mL of Dulbecco’s modified Eagle’s complete medium (DMEM, GIBCO, Grand Island, NY, United States) and incubated at 37°C. These cells were detached with 0.25% trypsinization and seeded in 6-well plates (1 × 10^5^ cells per well). The cells were pretreated with either compound C (50 μM) or solvent (DMSO) for 1.5 h and then incubated with/without TGF-β1 (10 ng/ml), WEL (10 μM) or solvents (PBS or DMSO) for 48 h. Then, these cells were subjected to the following analysis. In cell experiments, solutions of chemicals were prepared in DMSO, and diluted in FBS-free medium, the concentrations of DMSO is less than 0.05%.

The human type II alveolar epithelial cell MLE-12 were purchased from Saiqi BioTech Co., Ltd. (Shanghai, China) and maintained in DMEM/F12 (KeyGen BioTech Co., Ltd., Jiangsu, China) supplemented with 10% FBS (Hyclone, Thermo, South America), penicillin (100 U/mL) and streptomycin (100 μg/mL) at 37°C, with 95% humidity and 5% carbon dioxide. The cells were pretreated with either compound C (50 μM) or solvent for 1.5 h and then incubated with/without TGF-β1 (10 ng/ml), WEL (10 μM) or solvents for 48 h. Then, these cells were subjected to the following analysis.

### Cell Viability Assay

5 × 10^4^ cells were seeded in 96 well plates and incubated in DMEM or DMEN/F12 containing 10% FBS for 24 h. The cells were pretreated with either compound C (50 μM) or solvent (DMSO) for 1.5 h and subsequently incubated with/without TGF-β1 (10 ng/ml), WEL (10 μM) or solvent for 48 h, then MTT solvent (5 mg/ml) was added and incubated for 4 h at 37°C. The optical density was measured at 490 nm with 630 nm as reference wavelength.

### Animals

Male C57/BL6 mice (6–8 weeks old, weighing between 18 and 20 g) and male ICR mice (6–8 weeks old, weighing between 22 and 25 g) were supplied from Qinglongshan Standard Animal Propagation Center in Nanjing. The care and use of animals was performed in accordance with the General Recommendation and Provisions of the Chinese Experimental Animals Administration Legislation. All experiments were approved by the Institutional Ethical Committee of China Pharmaceutical University, Nanjing. Animals were housed in a climate-controlled room temperature at 22 ± 2°C and 50 ± 10% humidity with a 12 h light/dark cycle. Additionally, the animals were given free drinking water and conventional rodent chow.

### Mouse Model of BLM-Induced Pulmonary Fibrosis

The BLM-induced experimental pulmonary fibrosis model was described as our previous study (You et al., 2015). In brief, mice were divided into groups after 1 week of acclimation. Each group of mice was anesthetized with intraperitoneal injection of chloral hydrate solution (4%, 10 ml/kg) before intratracheal instillation, respectively, of BLM (5 mg/kg). Mice receiving an instillation of equivoluminal vehicle (0.9% sterilized saline solution) served as controls. Preliminary experimental were investigated in male ICR mice. We divide mice into five groups: normal group, BLM group, BLM and prednisone (PNS, positive drug),BLM and large dose of WEL (WEL-H, 10 mg/kg) as well as BLM and small dose of BLM groups (WEL-L, 2 mg/kg) at random. One week later after BLM administration, two doses of WEL (2 mg/kg or 10 mg/kg) and prednisone acetate (PNS, 6 mg/kg, positive drug) were orally administered to mice for 7 or 21 consecutive days, the control and the BLM groups were given the equivoluminal vehicle (0.9% sterilized saline). On the day 14 and day 28 after BLM instillation. After blood collection, each group’s mice were sacrificed randomly by excessive intraperitoneal injection of chloral hydrate. Lungs were excised for pulmonary coefficient measurement (lung weight/body weight; mg/g) (Turgut et al., 2016). The left lower lobes were fixed in 10% formalin for the examination of histopathology, and the other lung tissue samples were stored at -80°C.

Formal experiments were investigated in male C57/BL6 mice, 1 week later after BLM administration, WEL (10 mg/kg/day) were orally administered to mice for 14 consecutive days. On the day 21, mice were euthanized by excessive intraperitoneal injection of chloral hydrate. Lung tissues were excised for pulmonary index measurement (lung weight/body weight; mg/g). The left lower lobes were fixed in 10% formalin for the examination of histopathology, and the other lung tissue samples were stored at -80°C.

### Cytokine Assays in Bleomycin-Induced PF Model in ICR Mice

IL-1β, TNF-α, and TGF-β levels in lung tissues were measured with ELISA kits according to the instructions recommended by the manufactures (BioLegend, Inc., San Diego, CA, United States), and the optical density (OD) of the microplate was read at 450 nm.

### Histological Analysis

The lung tissues fixed with 10% formalin were embedded in paraffin for histological examination and stained with hematoxylin–eosin (HE) or Masson’s trichrome, then evaluated under a light microscopy conducted by experienced pathologists, who were blinded for groups. The results were scored in accordance with the previously reported method, and the score numbers (0–3) were, respectively, corresponded to the grades of -, +, ++, and +++ (Szapiel et al., 1979).

### Hydroxyproline Assay

Collagen deposition was determined by measuring the total HYP content, which was measured by a HYP assay kit according to the provided manufacturer’s protocol. In brief, lungs were hydrolyzed at 100°C for 40 min and mixed every 10 min. After neutralization with hydrochloric acid, the hydrolyzation products were diluted with distilled water, and assessed at 550 nm and expressed as μg/mg (You et al., 2015).

### Western Blot Analysis

The levels of Col I, α-SMA, TGF-β1, p-Smad2/3, p-AMPK, Raf1, MAPKs, Vimentin and E-cadherin were detected by Western blotting. Total proteins extracted from lung homogenate or cell lysate were lysed in ice-cold RIPA lysis buffer containing 1:100 dilution of phenylmethanesulfonyl fluoride (PMSF, Beyotime). Total protein concentrations were determined by BCA Protein Assay Kit (Beyotime). After boiling for 10 min, equal amounts of the protein (50 μg/lane) were separated by SDS-PAGE and transferred to PVDF membrane (Millipore, Billerica, MA, United States) that were probed with primary antibodies overnight at 4°C and HRP-labeled secondary antibodies at 25°C for 2 h and visualized using super ECL detection reagent (Beyotime).

### Preparation of RNA and RT-PCR Analysis

Total RNA from cultured cells and lung samples were isolated and one-step real-time RT-PCR and real-time PCR performed using SYBR Green PCR Reagents (TaKaRa, China), the StepOne^TM^ Real-Time PCR (Life Technologies, United States), and the Opticon DNA Engine (MJ Research Inc., South San Francisco, CA, United States). Total RNA was extracted from the treated cells or lung tissues using Trizol reagent (Invitrogen Life Technologies, United States), reverse-transcribed to complementary DNA (cDNA) using the TransScript first-Strand cDNA Synthesis kit (TOYOBO, Japan), and stored at -80°C until reverse transcription. The relative gene expression was quantified by Q-PCR using SYBR^®^ Premix Ex Taq^TM^ (TaKaRa, China) in StepOne^TM^ Real-Time PCR (Life Technologies, United States). In each reaction, 0.5 μg of total RNA was reverse transcribed before the following PCR conditions: 94°C for 2 min followed by 40 cycles at 94°C for 15 s, 58°C for 30 s, 72°C for 30 s, with final extension at 72°C for 10 min. Primers and amplicon sizes were shown in Table 1. The relative amount of mRNA was calculated using the comparative Ct (ΔCt) method compared with β-actin and expressed as the mean ± SD.

**Table 1:** Sequences of primers used for real-time quantitative PCR.

Gene	Forward primer (5′–3′)	Reverse primer (5′–3′)	Product size (bp)
M-α-SMA	CCA CGA AAC CAC CTA TAA CAG C	GGA AGG TAG ACA GCG AAG CC	236
M-Collagen I	CTG ACT GGA AGA GCG GAG AG	CGG CTG AGT AGG GAA CAC AC	116
M-TGF-β1	AGA GCC CTG GAT ACC AAC TAT TG	TGC GAC CCA CGT AGT AGA CG	286
M-Vimentin	TCC ACA CGC ACC TAC AGT CT	CCG AGG ACC GGG TCA CAT A	124
M-E-cadherin	CAG GTC TCC TCA TGG CTT TGC	CTT CCG AAA AGA AGG CTG TCC	175
M-β-actin	CTG AGA GGG AAA TCG TGC GT	CCA CAG GAT TCC ATA CCC AAG A	208
H-α-SMA	CTG TTC CAG CCA TCC TTC AT	TCA TGA TGC TGT TGT AGG TGG T	70
H-GAPDH	CAT CTT CTT TTG CGT CGC CA	TTA AAA GCA GCC CTG GTG ACC	115

### Statistical Analysis

Data were presented as mean ± SD from at least three independent experiments. One-way analysis of variance (ANOVA) was used for performing differences among different groups followed by the Student–Newman–Keuls test (GraphPad Prism Software 5.0, GraphPad Software Inc., San Diego, CA, United States). Values of *p* < 0.05 were considered statistically significant.

## Results

### WEL Protects Against Bleomycin-Induced Pulmonary Fibrosis in ICR Mice

**FIGURE 1:**
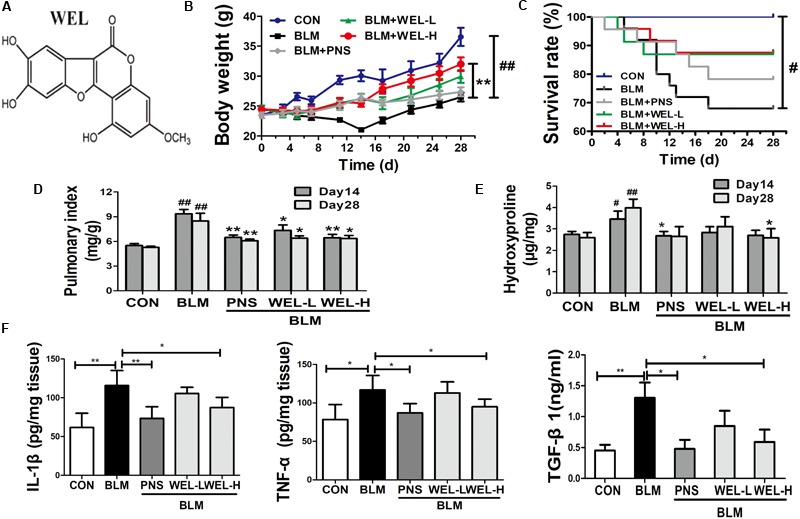
WEL attenuated bleomycin (BLM)-induced pulmonary fibrosis in ICR mice. One week after 5 mg/kg bleomycin (BLM) treatment, mice were orally administered with two doses of WEL-L (2 mg/kg/day) and WEL-H (10 mg/kg/day) and prednisone (PNS, 6 mg/kg) once a day for 7 or 21 days. **(A)** The chemical structure of WEL, changes of body weight **(B)**, survival rate **(C)**, and pulmonary index **(D)** were shown in different groups. **(E)** The HYP contents in lung tissues were determined by an assay kit. **(F)** The levels of pro-inflammatory cytokines (IL-1β, TNF-α, and TGF-β1) in lung tissue from different groups at day 14 were detected by ELISA assay. Data are shown as mean ± SD (*n* = 10). ^#^*p* < 0.05, ^##^*p* < 0.01 vs. the control group; ^∗^*p* < 0.05, ^∗∗^*p* < 0.01 vs. the BLM group.

Bleomycin-induced PF model in mice is characterized by activated myofibroblasts (Bhattacharyya et al., 2013). In this model, 7–9 days is the switch point from lung inflammation to fibrotic phase (Chaudhary et al., 2006). In the present study, two doses of WEL-L (2 mg/kg) and WEL-H (10 mg/kg) were orally administered for 14 days, respectively, starting 7 days after BLM (5 mg/kg) administration. The high dose of WEL treatment (WEL-H, 10 mg/kg) markedly attenuated BLM-induced the weight loss and the increasing pulmonary index as well as HYP content in lungs (Figure 1E,F). Moreover, the levels of pro-inflammatory cytokines, IL-1β, TNF-α, and TGF-β1, in lung tissues were elevated at day 14 from different groups, but greatly reduced after WEL treatment at the dose of 10 mg/k (Figure 1F), indicating an inhibitive effect of WEL in BLM-induced lung inflammation.

### WEL Protects Against Bleomycin-Induced Pathological Changes of Lungs in ICR Mice

**FIGURE 2:**
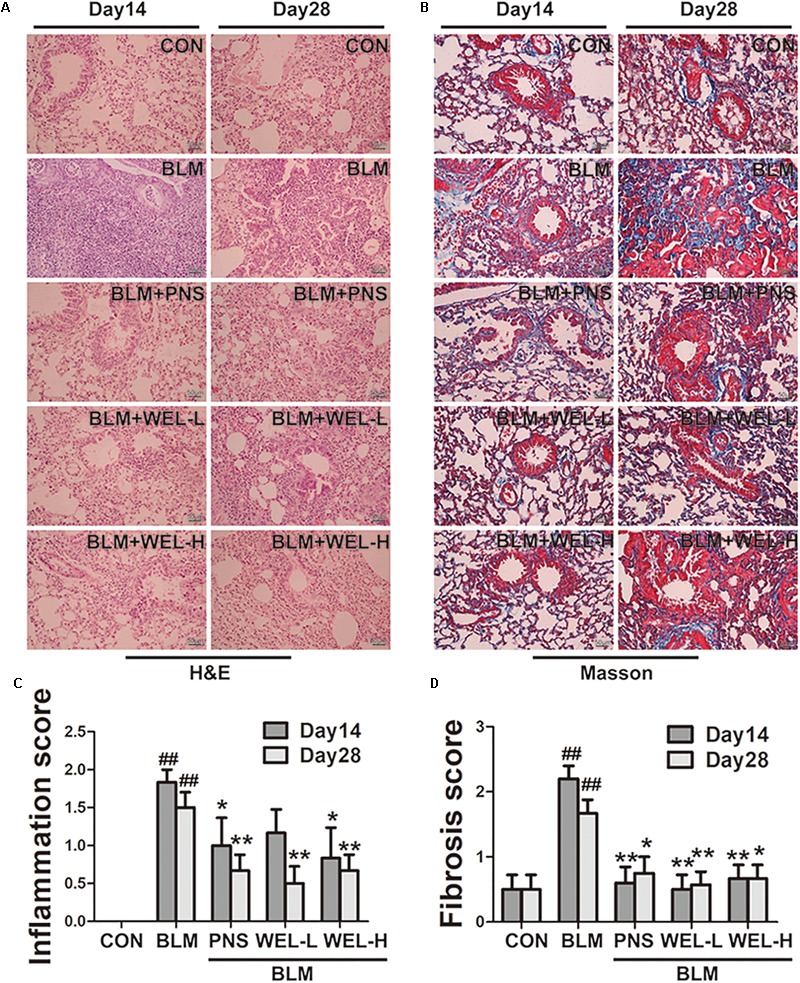
WEL protects against bleomycin-induced pathological changes of lungs in ICR mice. One week after BLM treatment (5 mg/kg), mice were orally administered with WEL-L (2 mg/kg) or WEL-H (10 mg/kg) and prednisone (6 mg/kg, positive drug) once a day for 7 or 21 days. Representative pictures (×200) of HE-stained **(A)** and Masson’s trichrome-stained **(B)** lung sections from mice on day 14 or day 28 were shown. Bar = 100 μm. The inflammation **(C)** and fibrosis **(D)** score numbers of 0–3, corresponding to the grades of –, +, ++, and +++, were evaluated by experienced pathologists in a blinded fashion. Data are presented as the mean ± SD (*n* = 10). ^##^*p* < 0.01 vs. the control group; ^∗^*p* < 0.05, ^∗∗^*p* < 0.01 vs. the BLM alone group.

The mice that received intratracheal instillations of BLM suffered serious lung damage and fibrosis, which manifested as weight loss, poor survival rate, collagen deposition in lung tissues. Histological analysis by HE and Masson’s staining showed WEL group displayed slightly thickened alveolar walls, some inflammatory cells, and minimum deposition of collagen fibers at day 14 and day 28 compared to BLM alone group (Figure 2).

### WEL Protects Against Bleomycin-Induced Pulmonary Fibrosis in C57/BL6 Mice

**FIGURE 3:**
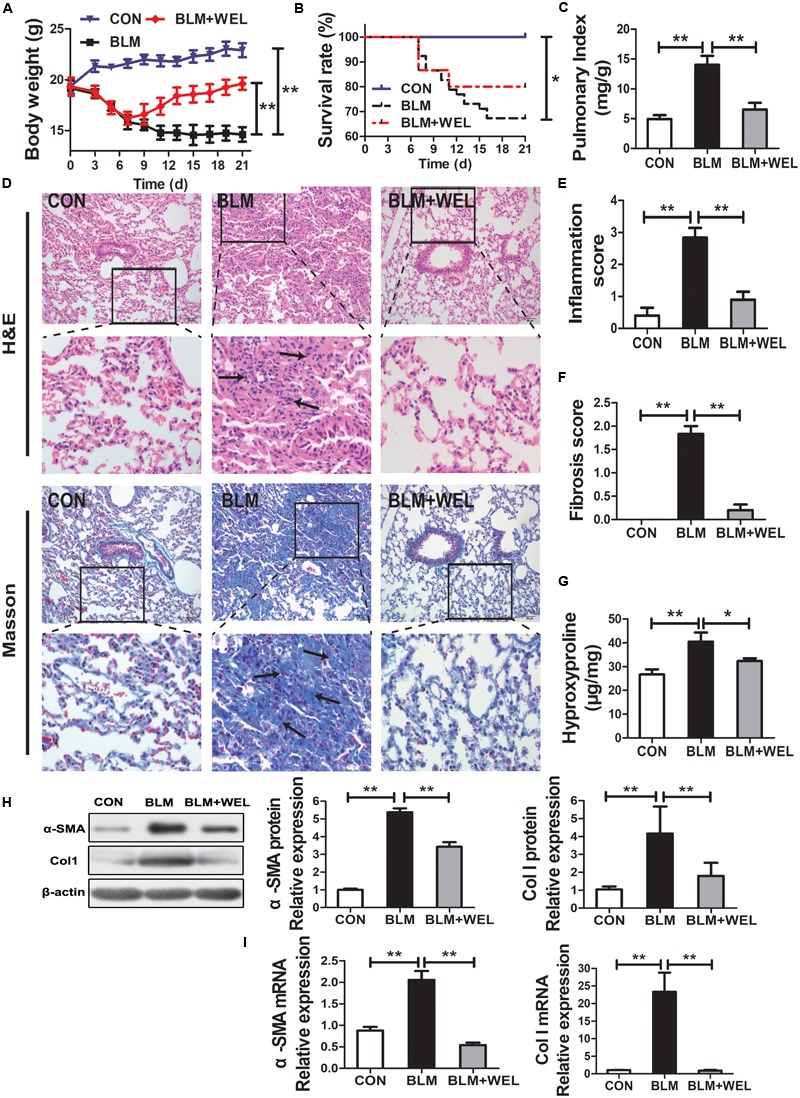
WEL ameliorated bleomycin (BLM)-induced pulmonary fibrosis in C57/BL6 mice. One week after 5 mg/kg BLM treatment, mice were orally administered with WEL (10 mg/kg) once a day for 14 days. **(A)** Body weight, **(B)** survival rate, and **(C)** pulmonary index of BLM mice and BLM mice that received WEL were determinated on day 21 (*n* = 6). **(D)** Representative pictures (×200) of HE-stained and Masson’s trichrome-stained lung sections from mice on day 21 were shown. Bar = 100 μm. The inflammation **(E)** and fibrosis **(F)** score numbers of 0–3, corresponding to the grades of –, +, ++, and +++, were evaluated by experienced pathologists in a blinded fashion. **(G)** HYP contents in lung tissues were determined by a assay kit. The protein expressions **(H)** of α-SMA and collagen I (Col I) in lung tissues were determined by Western blotting. The mRNA levels **(I)** of α-SMA and collagen I (Col I) in lung tissues were determined by PCR analysis. Data are presented as mean ± SD (*n* = 9). ^∗^*p* < 0.05, ^∗∗^*p* < 0.01.

In most studies, C57/BL6 mice are more susceptible to BLM-induced fibrosis (Lattaa et al., 2015). Then, C57/BL6 mice were also selected in the present study, and similar results were achieved in BLM-challenged PF model. The inflammation and fibrosis scores as well as HYP content in WEL groups were also significantly decreased compared to BLM alone group (Figure 3E–G). In addition, the expression of α-SMA (a hallmark of myofibroblasts) and Col I as well as its mRNA levels were also dramatically reduced in WEL-treated mice compared to BLM group (Figure 3H,I). Taken together, these results further confirmed that WEL could effectively ameliorated BLM-induced inflammation infiltration and fibrosis degree of lung tissues.

### WEL Down-Regulates TGF-β/Smad Signaling Pathway and Promotes the Activation of AMPK in Bleomycin-Induced PF in C57/BL6 Mice

**FIGURE 4:**
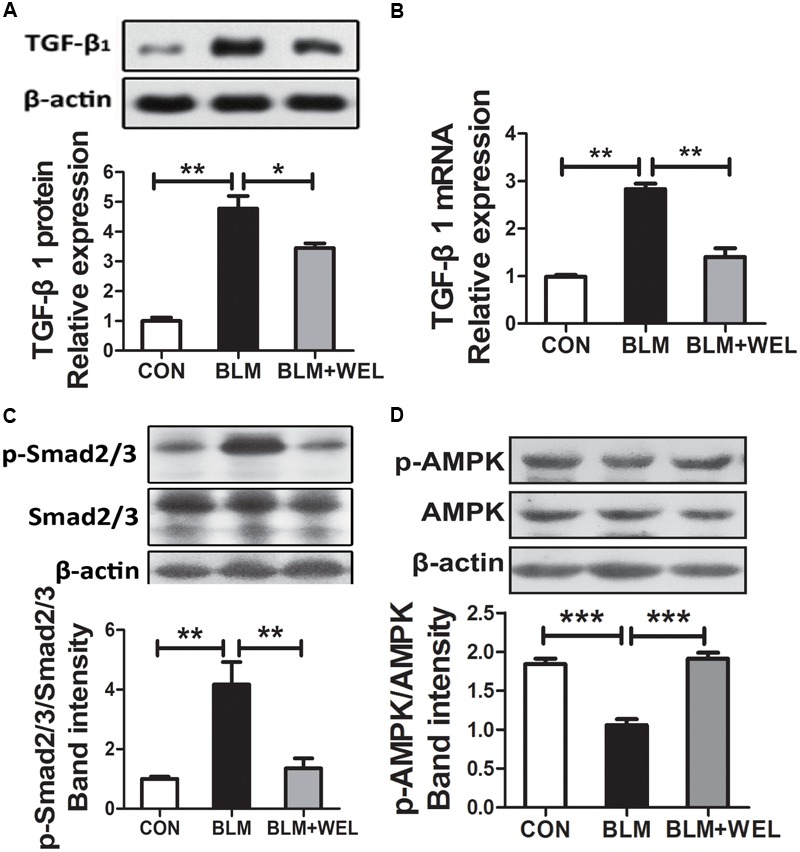
WEL regulated TGF-β/Smad signaling pathway and AMPK activation in lung tissue in bleomycin-induced PF in C57/BL6 mice. One week after 5 mg/kg BLM treatment, mice were orally administered with WEL (10 mg/kg) once a day for 14 days. The protein expression of TGF-β1 **(A)** and the phosphorylation levels of Smad2/3 **(C)** in lung tissues were determined by Western blotting. **(B)** The mRNA levels of TGF-β1 in lung tissues were determined by PCR analysis. **(D)** The protein phosphorylation levels of AMPK in lung tissues were determined by Western blotting. Data are presented as mean ± SD (*n* = 9). ^∗^*p* < 0.05, ^∗∗^*p* < 0.01, ^∗∗∗^*p* < 0.001.

Growth factor TGF-β1 has been widely detected in idiopathic pulmonary fibrosis, which activates fibroblast proliferation and collagen production, and TGF-β/Smad signaling pathway is the canonical signaling pathway during the fibrosis process (Pedram et al., 2010). As shown in Figure 4A,B, WEL treatment significantly decreased TGF-β1 over-expression and its mRNA levels as well as the phosphorylated Smad2/3 level in lungs compared to BLM alone group (Figure 4C). Additionally, WEL administration notably activated AMPK in lungs compared to BLM alone group (Figure 4D), suggesting that there is a close relationship between the activation AMPK by WEL treatment and its anti-fibrotic effects.

### WEL Prevents ECM Accumulation by Activating AMPK in PLFs Exposed to TGF-β1

**FIGURE 5:**
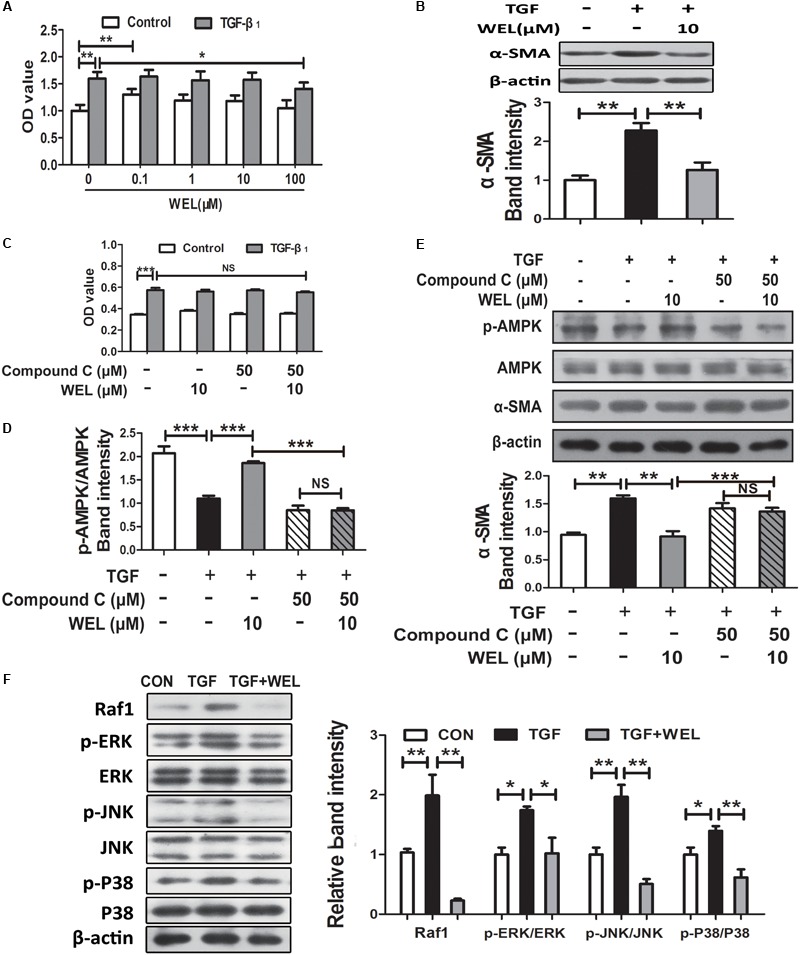
WEL ameliorated TGF-β-induced myofibroblast differentiation partly through Raf1-MAPK signaling pathway and AMPK activation in primary mouse lung fibroblasts (PLFs). The cells were pretreated with compound C (50 μM) or solvent for 1.5 h and subsequently incubated with/without TGF-β1 (10 ng/ml), WEL or solvent for 48 h. **(A)** The effect of WEL (0.1–100 μM) on PLFs proliferation cells were measured by the MTT assays. **(B)** The expression of α-SMA in PLFs treated with/without TGF-β1 was detected by Western blotting. **(C)** The inhibition of WEL or compound C on PLFs proliferation cells were measured by the MTT assays. **(D)** Expression of p-AMPK/AMPK in PLFs treated with/without TGF-β1 or compound C were determined by Western blotting. **(E)** The expression of α-SMA in PLFs treated with/without TGF-β1 or compound C were determined by Western blotting. **(F)** Protein expressions of Raf1, JNK/*p*-JNK, p38/*p*-p38, and ERK1/2/*p*-ERK1/2 in PLFs treated with/without TGF-β1 were detected by Western blotting. Data are presented as mean ± SD (*n* = 5). ^∗^*p* < 0.05, ^∗∗^*p* < 0.01, ^∗∗∗^*p* < 0.001. NS, non-significant.

Following lung injury, PLFs transform into myofibroblast-like cells and are the major source of ECM accumulation in the fibrotic lungs with α-SMA overexpression (Todd et al., 2012). In the current study, primary mouse lung fibroblasts (PLFs) were treated with TGF-β1 to induce fibrosis-related protein expression. As shown in Figure 5A, WEL at the concentration of 0.1–100 μM had no significant cytotoxicity to normal PFLs, but tend to weakly promote normal PFLs growths. Recent study reported that the activation of AMPK effectively alleviated inflammation-related fibrosis in lungs (Rangarajan et al., 2018).

Wedelolactone treatment (10 μM) significantly inhibited α-SMA overexpression (*P* < 0.01, Figure 5B), but the effect of WEL were significantly blocked by the inhibition of AMPK with compound C in TGF-β-stimulated PLFs (Figure 5E). In addition, TGF-β1 acts the non-genomic functions in lung myofibroblast proliferation via regulating Raf1-MAPK (ERK, JNK and P38) signaling pathways (Flores-Delgado et al., 2001). We found that WEL also significantly suppressed TGF-β-induced abnormal protein expressions of Raf1/MAPKs signaling pathways in PFLs (Figure 5F). Taken together, WEL treatment effectively suppressed the accumulation of ECM of activated lung fibroblasts partly by activating AMPK and its inflammation level.

### AMPK Activation by WEL Treatment Is Responsible for EMT Process

**FIGURE 6:**
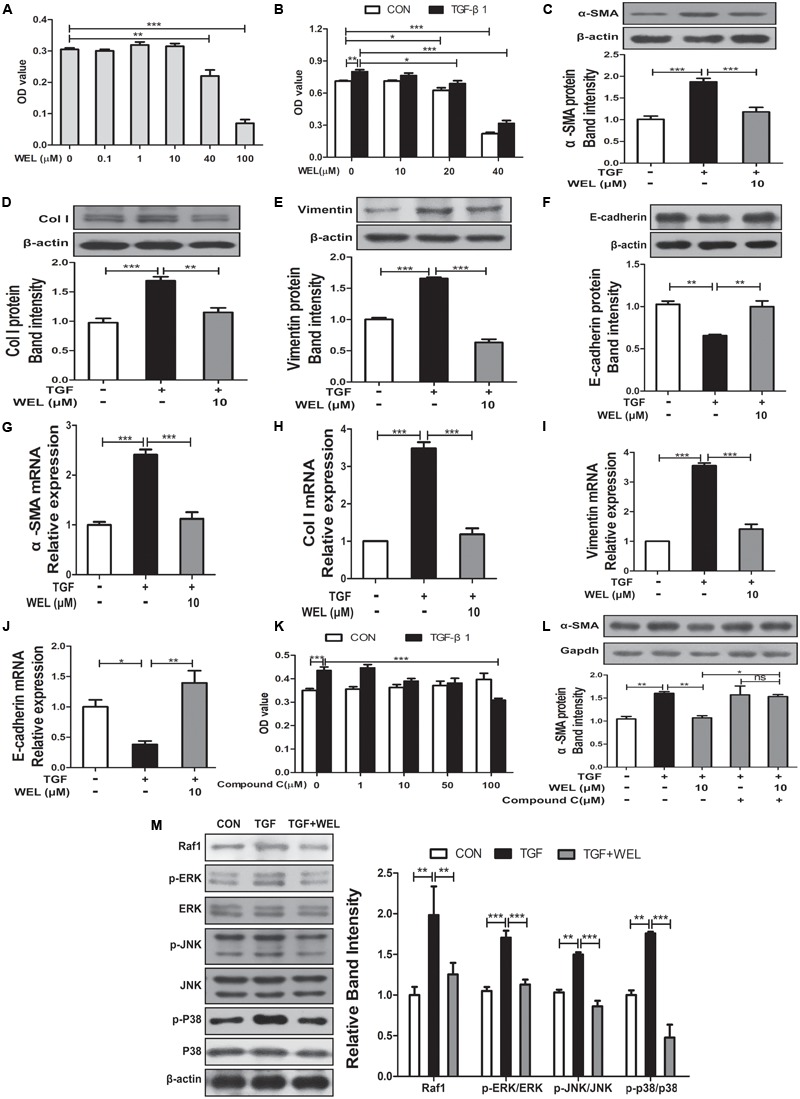
Regulation of WEL on the EMT of alveolar epithelial cells partly and its inflammation in TGF-β1 mediated MLE-12 cells. The cells were pretreated with compound C (50 μM) or solvent for 1.5 h and subsequently incubated with/without TGF-β1 (TGF, 10 ng/ml), WEL or solvent for 48 h. **(A,B)** Effects of WEL (0.1–100 μM) on proliferation cells were measured by the MTT assays. **(C–J)** Protein expressions and mRNA levels of α-SMA, Vimentin, Col I, and E-cadherin in MLE-12 cells treated with/without TGF-β1 were detected by Western blotting and PCR analysis. **(K)** Effect of compound C (0.1–100 μM) on proliferation cells were measured by the MTT assays. **(L)** The expression of α-SMA in MLE-12 cells treated with/without TGF-β1 or compound C were determined by Western blotting analysis. **(M)** Protein expressions of Raf1, JNK/*p*-JNK, p38/*p*-p38, and ERK1/2/p-ERK1/2 in MLE-12 treated with/without TGF-β1 were detected by Western blotting analysis. Data are presented as mean ± SD (*n* = 5). ^∗^*p* < 0.05, ^∗∗^*p* < 0.01, ^∗∗∗^*p* < 0.001. NS, non-significant.

TGF-β1 level mediated the epithelial-mesenchymal transition (EMT) process of alveolar epithelial cells during pulmonary fibrosis (Kuiper et al., 1998). In the present study, protective effects of WEL were obtained in TGF-β-treated MLE-12 cell lines. We examined the effect of WEL on cell viability at 0.1–100 μM for MLE-12 cells. WEL did not influence the cell growth at 0.1–10μM (Figure 6A,B). WEL treatment (10 μM) significantly inhibited the TGF-β1-induced abnormal expressions and mRNA levels of EMT markers, such as α-SMA, Vimentin, Col I and E-cadherin (Figure 6C–J) without influence on normal protein levels of MLE-12 cells (see Supplementary Figures S1, S2). However, the inhibition of WEL on EMT was significantly blocked by compound C (Figure 6K,L), suggesting that WEL could effectively ameliorated EMT of alveolar epithelial cells through activating AMPK. In addition, WEL also significantly inhibited Raf1-MAPKs signaling pathway in MLE-12 cells exposed to TGF-β1 (Figure 6M and Supplementary Figure S1). Taken together, WEL effectively suppressed EMT of alveolar epithelial cells partly through activating AMPK and its inflammation level.

## Discussion

Wedelolactone is the major component in Ecliptae Herba. WEL is reported to inhibit topoisomerase IIα and 5-lipoxygenase (Wagner and Fessler, 1986; Benes et al., 2011) hepatic stellate cells activation (Xia et al., 2013), induce cell apoptosis (Sarveswaran et al., 2012), activate G protein (Deng and Fang, 2012), protect bronchial epithelial cell (Ding et al., 2015) and attenuate carbon tetrachloride-induced liver injury in mice (Ping et al., 2012). The content of WEL in Ecliptae Herba is not less than 0.04% (g/g) recorded in China Pharmacopoeia (2015 editions). Previous study has confirmed that the ethanol extract of Ecliptae Herba effectively can attenuated BLM-induced pulmonary fibrosis (PF) in mice (You et al., 2015), but the protective effects of WEL on PF is unclear.

Pulmonary fibrosis is a progressive, fatal fibrosis disease without clear etiology (Adamson and Bowden, 1974). It is associated with higher mortality, weight loss and histopathological damage (infiltration of inflammatory cells, collapse of alveolar spaces, thickened alveolar wall and so on) in BLM-induced PF model. The capacity to ameliorate PF is associated with decreasing excessive collagen deposition, and the content of HYP is an indicator of collagen metabolism in connective tissue diseases (Liang et al., 2011). Previous trials to the PF treatment focused on inflammatory therapy, but researches indicate that the fibrosis process is driven by over-activated alveolar epithelial cells and fibroblasts (Daniels et al., 2004; Liang et al., 2011). During the process of EMT in lung fibrosis, epithelial cells disrupt their adhesion capacity due to the decrease in E-cadherin and the cytoskeleton rearrangement.

In the present study, the validity of WEL was investigated at the dosage of 2 mg/kg and 10 mg/kg, and WEL treatment at the dosage of 10 mg/kg showed significant protective effects on all kinds of clinical symptoms in BLM-induced lung fibrosis in ICR and C57/BL6 mice. Treatment with WEL at 10 mg/kg significantly reduced BLM-induced the collagen deposition in lung tissues compared to the model group (Figure 1, 3). Additionally, treatment with WEL also significantly impaired BLM-induced increase in TGF-β1 expression and Smad2/3 phosphorylation in the lungs (Figure 4). TGF-β/Smad is a canonical signaling pathway during fibrosis process which promotes myofibroblast differentiation and activates myofibroblasts to secrete excess amounts of ECM (King et al., 2011).

In present study, WEL treatment (10 mg/kg) significantly promoted the activation of AMPK in the lung tissues from BLM-treated mice (Figure 4). TGF-β-induced myofibroblast differentiation and BLM-induced lung fibrosis in mice were effectively inhibited by metformin-mediated AMPK activation (Li et al., 2015; Sato et al., 2016). *In vitro*, WEL effectively inhibited not only the accumulation of ECM in pro-inflammatory cytokine TGF-β-stimulated lung fibroblasts PLFs (Figure 5) but also the EMT of TGF-β1-mediated alveolar epithelial cells MLE-12 cells (Figure 6), however, these inhibition of WEL were significantly blocked by compound C (Figure 5, 6). AMPK activation is recognized to have potential beneficial effects on improving metabolic disorders and preventing organ dysfunction during fibrosis development (Zhao Y. et al., 2015), inhibiting AMPK activation by compound C (an AMPK inhibitor) could reverses metformin’s protective effects on lung fibrosis (Liu et al., 2014). Recent research also showed that WEL could activated AMPK in liver tissues from rat steatosis (Zhao J. et al., 2015).

The AMPK activator inhibits not only fibrosis (Flores-Delgado et al., 2001; Gong et al., 2005; Zhang et al., 2010) but also inflammation status (Langenbach et al., 2007; Sarveswaran et al., 2012). Moreover, TGF-β1 acts the non-genomic functions by MAPK kinase pathway, and estrogen inhibits lung myofibroblast proliferation via Raf1–MAPK (p38, ERK, and JNK) signaling pathway (Flores-Delgado et al., 2001). In the present study, we found that WEL treatments significantly suppressed TGF-β1-mediated inflammation station in activated lung fibroblasts and alveolar epithelial cells through down-regulating the activation of Raf1 and phosphorylated MAPKs (ERK, JNK, and p38) (Figure 5, 6). Previous studies have shown that WEL acts as phytoestrogen inhibits breast cancer cells by regulating ER genomic and non-genomic signaling pathways (Nehybova et al., 2015). However, further investigation, for example, whether the regulation of Raf1-MAPKs by WEL is an AMPK-dependent response or estrogen-like effect, is needed in the future. More comprehensive studies are needed to illustrate the precise molecular mechanism and the full effects of WEL on pulmonary fibrosis.

## Conclusion

In conclusion, WEL can decrease the associated inflammation by attenuating Raf1-MAPKs signaling pathway to inhibiting inflammatory cytokines production, and increase the activation of AMPK in the BLM-induced pulmonary fibrosis models, preventing an increase in pro-fibrotic markers such as Col I and α-SAM and attenuating a decreasing in anti-fibrotic marker such as E-cadherin. Mechanistic studies suggested that AMPK-mediated collagen suppression in particular is involved in WEL’s anti-fibrotic mechanisms. Additional investigations are necessary to elucidate the full anti-fibrotic potential of WEL as an effective therapy for PF patients, including that produced during BLM treatment.

## Author Contributions

J-yY, L-jT, BL, X-yY, and R-sL undertake main pharmacology experiments and help to Western blot analysis. H-fX has contributed to the preparation of WEL. C-fZ undertook the design of this project and did result analysis.

## Conflict of Interest Statement

The authors declare that the research was conducted in the absence of any commercial or financial relationships that could be construed as a potential conflict of interest.

## Abbreviations

α-SMA: α-smooth muscle actin
AMPK: adenosine 5′-monophosphate (AMP)-activated protein kinase
BLM: bleomycin
BSA: bovine serum albumin
COLI: collagen I
DAB: diaminobenzidine
ECL: enhanced chemiluminescence
ECM: extracellular matrix
EMT: epithelial mesenchymal transition
ERK: extracellular signal-regulated kinase
HYP: hydroxyproline
JNK: c-JUN N-terminal kinase
MAPK: mitogen-activated protein kinase
TGF-β1: transforming growth factor-β1
WEL: wedelolactone

## References

[B1] Adamson I. Y. Bowden D. H. (1974). The pathogenesis of bleomycin-induced pulmonary fibrosis in mice. *Am. J. Pathol.* 77 185–197. 4141224 PMC1910906

[B2] Benes P. Knopfova L. Trcka F. Nemajerova A. Pinheiro D. Soucek K. (2011). Inhibition of topoisomerase IIα: novel function of wedelolactone. *Cancer Lett.* 303 29–38. 10.1016/j.canlet.2011.01.002 21315506

[B3] Bhattacharyya S. Kelley K. Melichian D. S. Tamaki Z. Fang F. Su Y. Y. (2013). Toll-Like receptor 4 signaling augments transforming growth factor-(responses: a novel mechanism for maintaining and amplifying fibrosis in scleroderma. *Am. J. Pathol.* 182 192–205. 10.1016/j.ajpath.2012.09.007 23141927 PMC3538029

[B4] Chaudhary N. I. Schnapp A. Park J. E. (2006). Pharmacologic differentiation ofinflammation and fibrosis in the rat bleomycin model. *Am. J. Respir. Crit. Care Med.* 173 769–776. 10.1164/rccm.200505-717OC 16415276

[B5] Chichioco-Hernandez C. L. Paguigan N. D. (2010). Phytochemical profile of selected Philippine plants used to treat asthma. *Pharmacogn. J.* 2 198–202. 10.1016/S0975-3575(10)80092-6

[B6] Daniels C. E. Wilkes M. C. Edens M. Kottom T. J. Murphy S. J. Limper A. H. (2004). Imatinib mesylate inhibits the profibrogenic activity of TGF-β and prevents bleomycin-mediated lung fibrosis. *J. Clin. Invest.* 114 1308–1316. 10.1172/JCI200419603 15520863 PMC524221

[B7] Deng H. Fang Y. (2012). Anti-inflammatory gallic acid and wedelolactone are G protein-coupled receptor-35 agonists. *Pharmacology* 89 211–219. 10.1159/000337184 22488351

[B8] Ding S. Hou X. Yuan J. Tan X. Chen J. Yang N. (2015). Wedelolactone protects human bronchial epithelial cell injury against cigarette smoke extract-induced oxidant stress and inflammation responses through Nrf2 pathway. *Int. Immunopharmacol.* 29 648–655. 10.1016/j.intimp.2015.09.015 26411738

[B9] Flores-Delgado G. Bringas P. Buckley S. Anderson K. D. Warburton D. (2001). Nongenomic estrogen action in human lung myofibroblasts. *Biochem. Biophys. Res. Commun.* 283 661–667. 10.1006/bbrc.2001.4827 11341776

[B10] Gong L. K. Li X. H. Wang H. Zhang L. Chen F. P. Cai Y. (2005). Effect of Feitai on bleomycin-induced pulmonary fibrosis in rats. *J. Ethnopharmacol.* 96 537–544. 10.1016/j.jep.2004.09.046 15619575

[B11] Jahan R. Al-Nahain A. Majumder S. Rahmatullah M. (2014). Ethnopharmacological significance of *Eclipta alba* (L.) Hassk. (Asteraceae). *Int. Sch. Res. Notices* 2014:385969. 10.1155/2014/385969 27355071 PMC4897414

[B12] Jaiswal N. Bhatia V. Srivastava S. P. Srivastava A. K. Tamrakar A. K. (2012). Antidiabetic effect of *Eclipta alba* associated with the inhibition of alpha-glucosidase and aldose reductase. *Nat. Prod. Res.* 26 2363–2367. 10.1080/14786419.2012.662648 22348789

[B13] Khalil N. Parekh T. V. O’Connor R. Antman N. Kepron W. Yehaulaeshet T. (2001). Regulation of the effects of TGF-β1 by activation of latent TGF-β1 and differential expression of TGF-β receptors (TβR-I and TβR-II) in idiopathic pulmonary fibrosis. *Thorax* 56 907–915. 10.1136/thorax.56.12.907 11713352 PMC1745982

[B14] King T. E. Jr. Pardo A. Selman M. (2011). Idiopathic pulmonary fibrosis. *Lancet* 378 1949–1961. 10.1016/S0140-6736(11)60052-4 21719092

[B15] Kuiper G. G. Lemmen J. G. Carlsson B. Corton J. C. Safe S. H. Saag P. T. V. D. (1998). Interaction of estrogenic chemicals and phytoestrogens with estrogen receptor β. *Endocrinology* 139 4252–4263. 10.1210/endo.139.10.6216 9751507

[B16] Langenbach S. Y. Wheaton B. J. Fernandes D. J. Jones C. Sutherland T. E. Wraith B. C. (2007). Resistance of fibrogenic responses to glucocorticoid and 2-methoxyestradiol in bleomycin-induced lung fibrosis in mice. *Can. J. Physiol. Pharmacol.* 85 727–738. 10.1139/Y07-065 17823636

[B17] Lattaa V. D. Cecchettini A. Rya S. D. Morales M. A. (2015). Bleomycin in the setting of lung fibrosis induction: from biological mechanisms to counteractions. *Pharmacol. Res.* 97 122–130. 10.1016/j.phrs.2015.04.012 25959210

[B18] Ley B. Collard H. R. King T. E. Jr. (2011). Clinical course and prediction of survival in idiopathic pulmonary fibrosis. *Am. J. Respir. Crit. Care Med.* 183 431–440. 10.1164/rccm.201006-0894CI 20935110

[B19] Li L. Huang W. Li K. Zhang K. Lin C. Han R. (2015). Metformin attenuates gefitinib-induced exacerbation of pulmonary fibrosis by inhibition of TGF-beta signaling pathway. *Oncotarget* 6 43605–43619. 10.18632/oncotarget.6186 26497205 PMC4791254

[B20] Liang X. Tian Q. Wei Z. Liu F. Chen J. Zhao Y. (2011). Effect of Feining on bleomycin-induced pulmonary injuries in rats. *J. Ethnopharmacol.* 134 971–976. 10.1016/j.jep.2011.02.008 21333727

[B21] Liu W. Tan X. Sun H. Huang H. Jin P. Jia X. (2012). Protective effect and mechanism of *Ecliptae Herba* on cigarette smoke extract-induced cytotoxicity of NHBE cells. *Zhongguo Zhong Yao Za Zhi* 37 2444–2447. 23234146

[B22] Liu Y. Tang G. Li Y. Wang Y. Chen X. Gu X. (2014). Metformin attenuates blood-brain barrier disruption in mice following middle cerebral artery occlusion. *J. Neuroinflammation* 11:177. 10.1186/s12974-014-0177-4 25315906 PMC4201919

[B23] Manvar D. Mishra M. Kumar S. Pandey V. N. (2012). Identification and evaluation of anti Hepatitis C virus phytochemicals from *Eclipta alba*. *J. Ethnopharmacol.* 144 545–554. 10.1016/j.jep.2012.09.036 23026306 PMC3511619

[B24] Myllärniemi M. Kaarteenaho R. (2015). Pharmacological treatment of idiopathic pulmonary fibrosis-preclinical and clinical studies of pirfenidone, nintedanib, and N-acetylcysteine. *Eur. Clin. Respir. J.* 2 1–10. 10.3402/ecrj.v2.26385 26557253 PMC4629756

[B25] Nehybova T. Smarda J. Daniel L. Brezovsky J. Benes P. (2015). Wedelolactone induces growth of breast cancer cells by stimulation of estrogen receptor signalling. *J. Steroid. Biochem. Mol. Biol.* 152 76–83. 10.1016/j.jsbmb.2015.04.019 25934092

[B26] Pedram A. Razandi M. O’Mahony F. Lubahn D. Levin E. R. (2010). Estrogen receptor-β prevents cardiac fibrosis. *Mol. Endocrinol.* 24 2152–2165. 10.1210/me.2010-0154 20810711 PMC2958752

[B27] Ping P. Zhang C. F. Xu X. H. Zhang M. (2012). Effects of wedelolactone on mice’s acute hepatic injury induced by carbon tetrachloride. *Chin. Wild Plant Resour.* 31 41–43.

[B28] Postlethwaite A. E. Shigemitsu H. Kanangat S. (2004). Cellular origins of fibroblasts: possible implications for organ fibrosis in systemic sclerosis. *Curr. Opin. Rheumatol.* 16 733–738. 10.1097/01.bor.0000139310.77347.9c 15577612

[B29] Rangarajan S. Bone N. B. Zmijewska A. A. Jiang S. Park D. W. Bernard K. (2018). Metformin reverses established lung fibrosis in a bleomycin model. *Nat. Med.* 24 1121–1127. 10.1038/s41591-018-0087-6 29967351 PMC6081262

[B30] Roy R. K. Thakur M. Dixit V. K. (2008). Hair growth promoting activity of *Eclipta alba* in male albino rats. *Arch. Dermatol. Res.* 300 357–364. 10.1007/s00403-008-0860-3 18478241

[B31] Sarveswaran S. Gautam S. C. Ghosh J. (2012). Wedelolactone, a medicinal plant-derived coumestan, induces caspase-dependent apoptosis in prostate cancer cells via downregulation of PKC𝜀 without inhibiting Akt. *Int. J. Oncol.* 41 2191–2199. 10.3892/ijo.2012.1664 23076676 PMC3541032

[B32] Sato N. Takasaka N. Yoshida M. Tsubouchi K. Minagawa S. Araya J. (2016). Metformin attenuates lung fibrosis development via NOX4 suppression. *Respir. Res.* 17 107–189. 10.1186/s12931-016-0420-x 27576730 PMC5006432

[B33] Selman M. Pardo A. (2001). Idiopathic pulmonary fibrosis: an epithelial/fibroblastic cross-talk disorder. *Respir. Res.* 3:3. 11806838 10.1186/rr175 PMC64814

[B34] Sharma J. Gairola S. Gaur R. D. Painuli R. M. (2012). The treatment of jaundice with medicinal plants in indigenous communities of the Sub-Himalayan region of Uttarakhand. *India J. Ethnopharmacol.* 143 262–291. 10.1016/j.jep.2012.06.034 22759701

[B35] Sime P. J. Xing Z. Graham F. L. Csaky K. G. Gauldie J. (1997). Adenovector-mediated gene transfer of active transforming growth factor-beta1 induces prolonged severe fibrosis in rat lung. *J. Clin. Invest.* 100 768–776. 10.1172/JCI119590 9259574 PMC508247

[B36] Szapiel S. V. Elson N. A. Fulmer J. D. Hunninghake G. W. Crystal R. G. (1979). Bleomycin-induced interstitial pulmonary disease in the nude, athymic mouse. *Am. Rev. Respir. Dis.* 120 893–899. 92208 10.1164/arrd.1979.120.4.893

[B37] Tabassum N. Agrawal S. S. (2004). Hepatoprotective activity of *Eclipta alba* Hassk. against paracetamol induced hepatocellular damage in mice. *JK Pract.* 11 278–280.

[B38] Tewtrakul S. Subhadhirasakul S. Tansakul P. Cheenpracha S. Karalai C. (2011). Antiinflammatory constituents from *Eclipta prostrata* using RAW264.7 macrophage cells. *Phytother. Res.* 25 1313–1316. 10.1002/ptr.3383 21312307

[B39] Todd N. W. Luzina I. G. Atamas S. P. (2012). Molecular and cellular mechanisms of pulmonary fibrosis. *Fibrog. Tissue Repair* 5:11. 10.1186/1755-1536-5-11 22824096 PMC3443459

[B40] Turgut N. H. Kara H. Elagoz S. Deveci K. Gungor H. Arslanbas E. (2016). The protective effect of naringin against bleomycin-induced pulmonary fibrosis in Wistar rats. *Pulm. Med.* 2016:7601393. 10.1155/2016/7601393 26977316 PMC4764747

[B41] Wagner H. Fessler B. (1986). In vitro 5-lipoxygenase inhibition by Elipta alba extracts and the coumestan derivative wedelolactone. *Planta Med.* 52 374–377. 10.1055/s-2007-969189 3797501

[B42] Wynn T. A. (2011). Integrating mechanisms of pulmonary fibrosis. *J. Exp. Med.* 208 1339–1350. 10.1084/jem.20110551 21727191 PMC3136685

[B43] Xia Y. Z. Chen J. Cao Y. Xu C. S. Li R. M. Pan Y. (2013). Wedelolactone exhibits anti-fibrotic effects on human hepatic stellate cell line LX-2. *Eur. J. Pharmacol.* 714 105–111. 10.1016/j.ejphar.2013.06.012 23791612

[B44] You X. Y. Xue Q. Fang Y. Liu Q. Y. Zhang C. F. Zhao C. (2015). Preventive effects of *Ecliptae Herba* extract and its component, ecliptasaponin A, on bleomycin-induced pulmonary fibrosis in mice. *J. Ethnopharmacol.* 175 172–180. 10.1016/j.jep.2015.08.034 26385580

[B45] Zhang C. F. Sun Z. H. Zhang D. Zhang M. (2010). Sulphur compounds from the aerial parts of *Eclipta prostrata*. *Biochem. Syst. Ecol.* 38 1253–1256. 10.1016/j.bse.2010.12.024 25443644

[B46] Zhao J. Miyamoto S. You Y. H. Sharma K. (2015). AMP-activated protein kinase (AMPK) activation inhibits nuclear translocation of Smad4 in mesangial cells and diabetic kidneys. *Am. J. Physiol. Renal Physiol.* 308 F1167–F1177. 10.1152/ajprenal.00234.2014 25428125 PMC4437003

[B47] Zhao Y. Peng L. Yang L. Xu X. Li W. Luo X. (2015). Wedelolactone regulates lipid metabolism and improves hepatic steatosis partly by AMPK activation and up-regulation of expression of PPARα/LPL and LDLR. *PLoS One* 10:e0132720. 10.1371/journal.pone.0132720 26168156 PMC4500417

