# Supplementary material for: Wedelolactone Attenuates Pulmonary Fibrosis Partly Through Activating AMPK and Regulating Raf-MAPKs Signaling Pathway
Source: Front Pharmacol. 2019 Mar 5;10:151. doi: 10.3389/fphar.2019.00151 (PMC6411994; doi:10.3389/fphar.2019.00151)
Supplement: Supplementary file 1 [file Table_1.pdf]

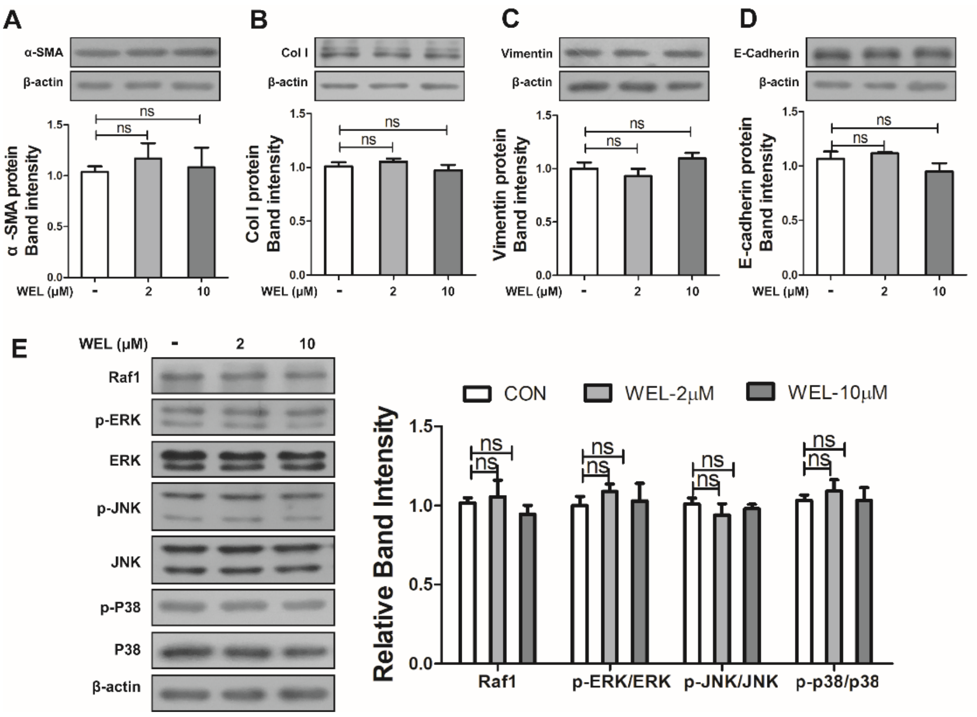

Fig. S1 Influence of WEL on related protein lelves ofnormal alveolar epithelial MLE-12 cells. The cells were incubated with WEL(2 or 10μM)or solvent for 48h. (A-D) Protein expressionsofα-SMA, Vimentin, Col I and E-cadherin in MLE-12 cells weres detected by western blotting and PCR analysis. (E) Protein expressions of Raf1, JNK/p-JNK, p38/ p-p38, and ERK1/2 /p-ERK1/2 were detected by western blotting analysis. Data are presented as mean ±SD (n = 5). NS, non-significant.

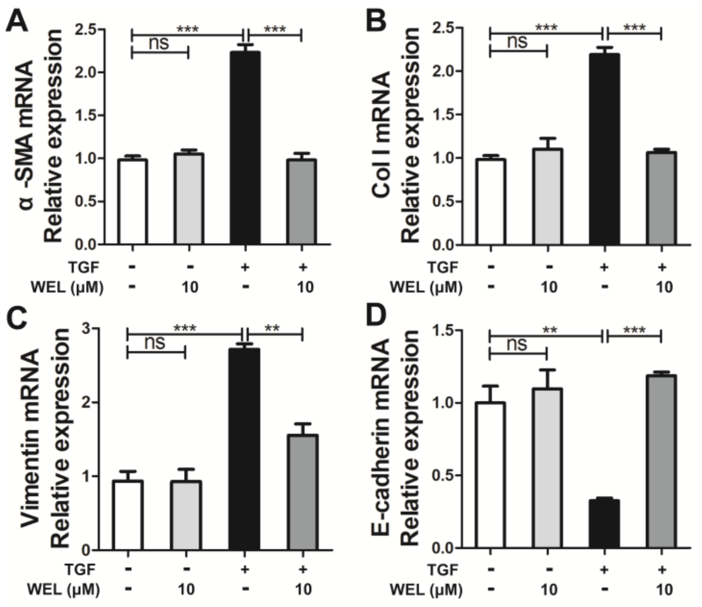

Fig.S2 Regulation of WEL on the EMT of alveolar epithelial cells in TGF-β1 mediatedMLE-12 cells.(A-D) mRNA levelsofα-SMA, Col I, Vimentin, and E-cadherin in MLE-12 cells treated with/without TGF-β1weres detected by PCR analysis. Data are presented as mean ± SD (n = 5). NS, non-significant.*p < 0.05, **p < 0.01, ***p < 0.001.NS, non-significant.
